# A Spatial Analysis of Access to Physical Activity Infrastructure and Healthy Food in Regional Tasmania

**DOI:** 10.3389/fpubh.2021.773609

**Published:** 2021-12-01

**Authors:** Sisitha Jayasinghe, Emily J. Flies, Robert Soward, Dave Kendal, Michelle Kilpatrick, Timothy P. Holloway, Kira A. E. Patterson, Kiran D. K. Ahuja, Roger Hughes, Nuala M. Byrne, Andrew P. Hills

**Affiliations:** ^1^College of Health and Medicine, University of Tasmania, Hobart, TAS, Australia; ^2^School of Natural Sciences, University of Tasmania, Hobart, TAS, Australia; ^3^Healthy Landscapes Research Group, University of Tasmania, Hobart, TAS, Australia; ^4^School of Geography, Planning and Spatial Sciences, University of Tasmania, Hobart, TAS, Australia

**Keywords:** obesity, physical activity, food environment, spatial analysis, regional area, NW Tasmania, Regional Australia

## Abstract

Prevalence of physical inactivity and obesity continues to increase in regional areas such as North-West (NW) Tasmania and show no signs of abating. It is possible that limited access to physical activity infrastructure (PAI) and healthier food options are exacerbating the low levels of habitual physical activity and obesity prevalence in these communities. Despite a burgeoning research base, concomitant exploration of both physical activity and food environments in rural and regional areas remain scarce. This research evaluated access (i.e., coverage, variety, density, and proximity) to physical activity resources and food outlets in relation to socioeconomic status (SES) in three NW Tasmanian communities. In all three study areas, the PAI and food outlets were largely concentrated in the main urban areas with most recreational tracks and natural amenities located along the coastline or river areas. Circular Head had the lowest total number of PAI (*n* = 43) but a greater proportion (30%) of free-to-access outdoor amenities. There was marked variation in accessibility to infrastructure across different areas of disadvantage within and between sites. For a considerable proportion of the population, free-to-access natural amenities/green spaces and recreational tracks (73 and 57%, respectively) were beyond 800 m from their households. In relation to food accessibility, only a small proportion of the food outlets across the region sells predominantly healthy (i.e., Tier 1) foods (~6, 13, and 10% in Burnie, Circular Head and Devonport, respectively). Similarly, only a small proportion of the residents are within a reasonable walking distance (i.e., 5–10 min walk) from outlets. In contrast, a much larger proportion of residents lived close to food outlets selling predominantly energy-dense, highly processed food (i.e., Tier 2 outlets). Circular Head had at least twice as many Tier 1 food stores per capita than Devonport and Burnie (0.23 vs. 0.10 and 0.06; respectively) despite recording the highest average distance (4.35 and 5.66 km to Tier 2/Tier 1 stores) to a food outlet. As such, it is possible that both food and physical activity environment layouts in each site are contributing to the obesogenic nature of each community.

## Introduction

Physical inactivity and sub-optimal dietary practises are global public health concerns, and inexorably linked to poor health outcomes (1, 2). Sustained inactivity impacts short- and long-term health and quality of life, with substantial attendant economic costs (3). In Australia, as is the case in many developed nations, low levels of physical activity are very common; 55% of Australians aged 18 years and over do not engage in recommended levels of habitual physical activity (4). Similarly, consumption of fruit and vegetables—an important indicator of a healthy diet and lifestyle—is low, with the vast majority of Australian adults failing to meet national dietary recommendations. These patterns of low levels of physical activity and sub-optimal dietary practises are particularly acute in regional areas such as Northwest (NW) Tasmania which report some of the lowest levels of physical activity in the country (5). Moreover, only 3% of Tasmanian adults meet the National Health and Medical Research Council guidelines for fruit and vegetable consumption (5).

The built environment—broadly defined as major physical structures and facilities where people live, work, and play, such as buildings, stores, streets, homes, schools, parks, playgrounds, and other infrastructure (e.g., fitness and community centres)—have a significant impact on the physical activity and dietary patterns of individuals (6, 7). The built environment differs notably between urban and regional settings, with major downstream effects on health outcomes (8, 9). For instance, lack of physical activity resources and limited access to healthy food options in regional areas can manifest as higher prevalence of lifestyle-related chronic diseases (10–12). The significantly higher prevalence of obesity and associated chronic diseases in adults living in the NW of Tasmania is a good case in point.

Economic precarity in most regional locations further complicates the relationship between the built environment and health. There is strong evidence for the association between socioeconomic status (SES) and obesity (13–17). Regional NW Tasmania is a relatively disadvantaged area (18, 19), and this may partly explain the prevalence of obesity and associated chronic disease. Physical activity resources and access to healthy food may also be inequitably distributed in these locations and reinforce patterns of obesity. The links between the built environment and PA/PI are manifold and complex. Nevertheless, the availability of Geographic Information System (GIS) based measures such as population density, land-use mix, access to recreational facilities, and street connectivity etc., and a multitude of observational infrastructure assessment techniques in recent times, has demonstrably improved the ability to assess the capacity of the built environment and its effect on health and wellbeing (20–22).

A considerable body of empirical evidence illustrates associations between obesity, SES, and attributes of the built environment, including access to healthy food and physical activity resources (23–25). However, to date, most of this evidence has been generated in large urban areas. A better understanding of the local food and physical activity environment in regional NW Tasmania may elucidate some of the drivers of high rates of obesity and chronic disease in this and similar regional areas, and potential intervention points to improve health in these locations. Therefore, this research aimed to evaluate access (i.e., coverage, variety, density, and proximity) to physical activity resources and healthier food in NW Tasmania. Secondly, we evaluated the relationship between access to physical activity opportunities, healthier food, population density, and SES in the region.

## Materials and Methods

**Figure 1:**
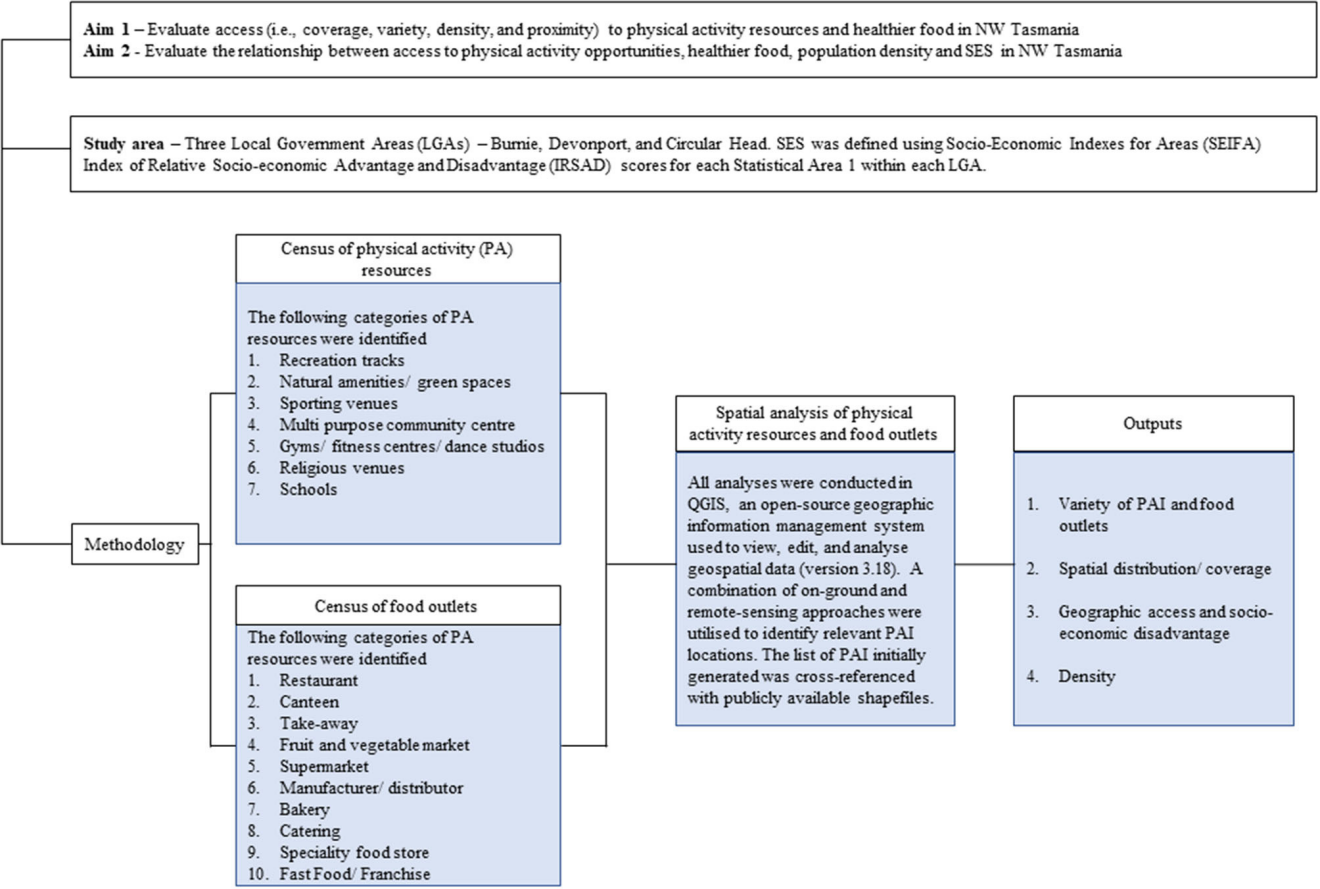
Flow chart of study procedures.

A visual representation of study procedure is presented in Figure 1.

### Study Area

The study regions included three Local Government Areas (LGAs)—Burnie, Devonport, and Circular Head—in the NW of Tasmania. Briefly, the selected LGAs were classified as Remoteness Area 2 (Inner Regional Australia) and 3 (Outer Regional Australia), according to the Australian Statistical Geography Standard classification system and contained sufficiently demarcated administrative boundaries from neighbouring communities. SES was defined using Socio-Economic Indexes for Areas (SEIFA) Index of Relative Socio-economic Advantage and Disadvantage (IRSAD) scores for each Statistical Area 1 (SA1; ~400 people) within each LGA, accessed via the Australian Bureau of Statistics (26).

### Census of Physical Activity Resources and Food Outlets

Seven categories [based on previous literature (27)] of physical activity infrastructure/ resources (referred to herein as PAI) was generated using an extensive online search via Google, Yellow Pages, Facebook, Local Council and State Government web pages. Subsequently, two categories of PAI (Recreation track and Natural amenity/green space) were further classified as “free-to-access” based on them being accessible around the clock with no associated monetary costs.

1. Recreation track: free-to-access, purpose-built outdoor pathway, trail, or track for recreational physical activity.
2. Natural amenities, green space: free-to-access area of vegetation or a beach, set apart for recreational purposes. National parks were not included in this category as they require a permit to visit them.
3. Sporting venues: purpose-built outdoor sporting areas for organised sport.
4. Multi-purpose community centre: purpose-built structure for informal, formal, and social recreational and physical activities.
5. Gyms/fitness centres/dance studios: purpose-built structures for strength and conditioning activities and learning/rehearsing dance.
6. Religious venues (e.g., churches): purpose-built structures (primarily) for public religious activity with secondary usage for recreational pursuits.
7. Schools: purpose-built structures for educating children which also have outdoor play equipment.

Ten categories of food outlets were initially identified through business registration lists (obtained via LGA Environmental Health Officers) for each study site. These were subsequently cross-referenced and confirmed through online verification by trained research personnel.

1. Restaurant - Seated venue where food is purchased and primarily eaten onsite.
2. Canteen - Where food is prepared / served and associated with a school, aged care, or sporting facility site.
3. Take Away - Where food is prepared and purchased to take away.
4. Fruit & Vegetable Market - Primarily sells fruit and vegetables.
5. Supermarket - A primarily self-service shop selling foods and household goods.
6. Manufacturer/distributor - Manufacturers or processes food that is on sold mainly to other businesses for resale (could be home based or larger commercial operation).
7. Bakery - Produces baked goods / bakery products.
8. Catering - Mobile business that provides prepared food (e.g., food vans, caterers that cater for events, service /special interest clubs etc.).
9. Specialty food store - Butcher or fishmonger.
10. Fast Food/ Franchise - Business belong to franchise and sells fast food primarily to take away.

For analytical purposes, food outlets were stratified into “Tier 1” (green grocers, butchers, supermarkets and health food shops etc.), and “Tier 2” (chain and non-chain fast-food outlets, bakeries, sweet food retailers, and convenience stores, etc.) outlets (refer to **Table 2** for all categories and their designations) using previously published approaches (23, 28, 29). Briefly, a combination of visibility of fruit and vegetables in the outlet consumer view-space (as judged by research team members) and the level of “food processing” (defined as all methods and techniques utilised by food, drink, and associated industries to convert fresh foods into food products) was considered in the assignment of outlets to one of two categories.

### Spatial Analysis

All analyses were conducted in QGIS, an open-source geographic information management system used to view, edit, and analyse geospatial data (version 3.18) (30). A combination of on-ground and remote-sensing approaches were utilised to identify relevant PAI locations. The list of PAI initially generated was cross-referenced with publicly available shapefiles (i.e., Public Land Classification, Local Government Authority Reserves, Tasmanian Reserve Estates and Infrastructure and Utilities to identify Track and Ferry Routes) to further identify potentially relevant natural amenities/green space and recreational tracks and their spatial extents. Subsequently, the potentially relevant tracks and polygons (natural amenities) were “ground trothed” using local knowledge; irrelevant sites were removed, and spatial extents corrected as needed.

Once the final layers of relevant points, lines and polygons were formed, population-weighted centroids were created for each SA1/suburb in the area using the 2019 average annual night-time lights (31). Unpopulated SA1s were removed from all analyses (*n* = 6). Weighted centroids were used to calculate the average distance to the nearest PAI or Tier 1 food outlet using the point-to-point calculations tool (for points) and the NNjoin tool for distance to nearest polygon or line.

The number of points per LGA was calculated by “Count points in a polygon” tool for PAI points, healthy and Tier 2 food sites. Length of tracks/trails was calculated using the vector analysis “sum line lengths” tool. Buffers were created for each line, point and polygon by first converting these shapefiles into the Lambert projection system, and then adding the 400 and 800 m buffers—~5 and 10 min of walking distance (32, 33)—for each geometry type. SA1s were intersected with the buffered areas and the percent of the area of the SA1 inside the buffer was used to calculate the percent of the population with access to that PAI. These values were calculated for each SA1 and averaged across IRSAD rankings or population density quintiles for each region of interest.

## Results

### Variety of PAI and Food Outlets

All three study sites were found to have a variety of PAI food outlets (Tables 1, 2). Circular Head had the lowest number of PAI (*n* = 43) although 30% were free-to-access outdoor amenities (i.e., natural amenities/green space and recreational tracks)—the highest proportion in the wider study region. Approximately one quarter of all PAI in the study sites are sporting venues (Table 1). It is also noteworthy that ~20% of PAI are located within school premises (Table 1). Despite the variability, ~6, 13, and 10% of the food outlets in Burnie, Circular Head and Devonport, respectively, were classified as “Tier 1” (Table 2). Further, there was a noticeable absence of “fast food outlets” in Circular Head and “speciality food stores” in Burnie and Devonport (Table 2).

**Table 1:** Proportion of physical activity infrastructure (PAI) in Burnie, Circular Head, and Devonport.

	**Burnie**	**Circular Head**	**Devonport**
	**% (*n*)**	**% (*n*)**	**% (*n*)**
Recreation track	5% (3)	7% (3)	14% (12)
Natural amenities/green space	16% (12)	23% (18)	12% (9)
Sporting venues	24% (14)	23% (8)	26% (16)
Multi-purpose community centre	2% (2)	7% (3)	4% (3)
Gyms/fitness centres/dance studios	21% (13)	5% (2)	14% (19)
Churches	8% (6)	16% (8)	13% (10)
PAI in schools	24% (15)	19% (8)	18% (14)
Total PAI	100%	100%	100%
	(*N* = 62)	(*N* = 43)	(*N* = 78)

**Table 2:** Variety of Tier 1/Tier 2 food outlets in Burnie, Circular Head, and Devonport.

	**Burnie**	**Circular Head**	**Devonport**
	***N* = 163**	***N* = 119**	***N* = 248**
Tier 1 food options			
Fruit and vegetable market	1	1	1
Supermarket	9	10	13
Specialty food store	0	0	8
Tier 1 manufacturer/distributor	0	5	4
Total Tier 1 food outlets	10	16	26
Tier 2 food options			
Restaurant	51	33	101
Canteen	37	28	40
Take away	35	11	18
Bakery	11	3	3
Catering	0	10	25
Fast food/Franchise	14	0	13
Tier 2 manufacturer/Processor	5	18	23
Total Tier 2 food outlets	153	103	222
Proportion of Tier 1 (*n*) from total	6% (10/163)	13% (16/119)	10% (26/248)

### Spatial Distribution/Coverage

**Figure 2:**
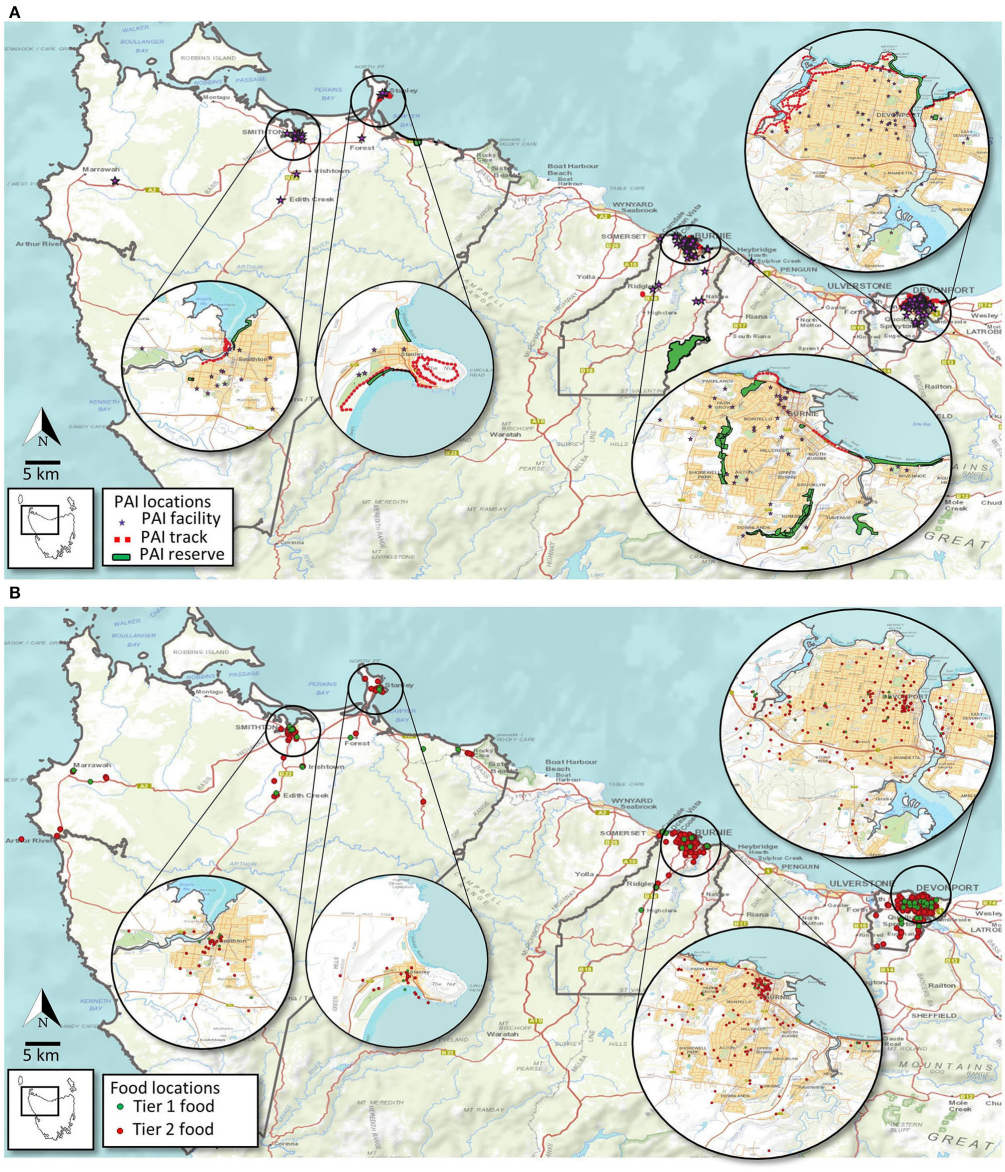
Spatial distribution of PA infrastructure **(A)** and food outlets **(B)** across the study area.

Geographical locations of the identified PAI and food outlets in Burnie, Circular Head and Devonport are presented in Figures 2A,B. In all three study areas, the PAI (Figure 2A) and food (Figure 2B) were largely concentrated in the main urban areas (see insets) with most recreational tracks and natural amenities (e.g., beaches) located along the coastline or the riverine areas. In all locations, non-free-to-access PAI points (which included Categories 3–7) were more distributed throughout the urban area than the tracks or natural amenities; however, Burnie has several inland natural amenities/green spaces within the urban area and beyond (Figure 2A). The number of “Tier 2” food outlets vastly out-weigh the “Tier 1” food outlets in all 3 study areas (Figure 2B) with no distinct spatial distribution pattern observable. The availability of PAI points per 100 people ranged between 0.2 and 0.4 across the sites with Circular Head recording the highest PAI points per capita. The amount (in km^2^) of natural amenities per 100 people was low across the three regions, with Devonport having <0.01 km^2^ per 100 capita (Table 3). Further, length of walking track per capita is substantially lower in Burnie compared with the other 2 sites (Table 3). Devonport has the most accessible PAI (average distance <2 km) while residents in Circular Head were, on average, ~8 km away from the nearest PAI (Table 3).

**Table 3:** Summary of the availability of physical activity infrastructure (PAI) and food outlets per region.

**Region**	**Number of PAI points per 100 people**	**Area of natural amenity/green space per 100 people (km^**2**^)**	**Length of recreational track per 100 people (km)**	**Healthy food outlets per 100 people**	**Avg distance to PAI point (km)**	**Avg distance to natural amenity/green space (km)**	**Avg distance to recreational track (km)**	**Avg distance to Tier 2 food outlet (km)**	**Avg distance to Tier 1 food outlet (km)**
Burnie	0.26	0.11	0.16	0.06	3.88	4.27	6.10	4.14	4.48
Circular Head	0.39	0.03	1.09	0.23	8.79	8.04	11.79	4.35	5.66
Devonport	0.23	0.00	0.85	0.10	1.80	3.41	2.76	0.95	1.82
Study region	0.27	0.05	0.63	0.11	3.73	4.47	5.52	2.79	3.50

As for the food outlets, Circular Head had at least twice as many Tier 1 food stores per capita than Devonport and Burnie (0.23 vs. 0.10 and 0.06; respectively) despite recording the highest average distance (4.35 and 5.66 km to Tier 2/Tier 1 stores) to a food outlet (Table 3) and the lowest number of food outlets overall (Table 2). Proximity to food outlets was highest in Devonport with a Tier 2 food outlet available in less than a kilometre from the average resident (Table 3).

### Geographic Access and Socio-Economic Disadvantage

**Figure 3:**
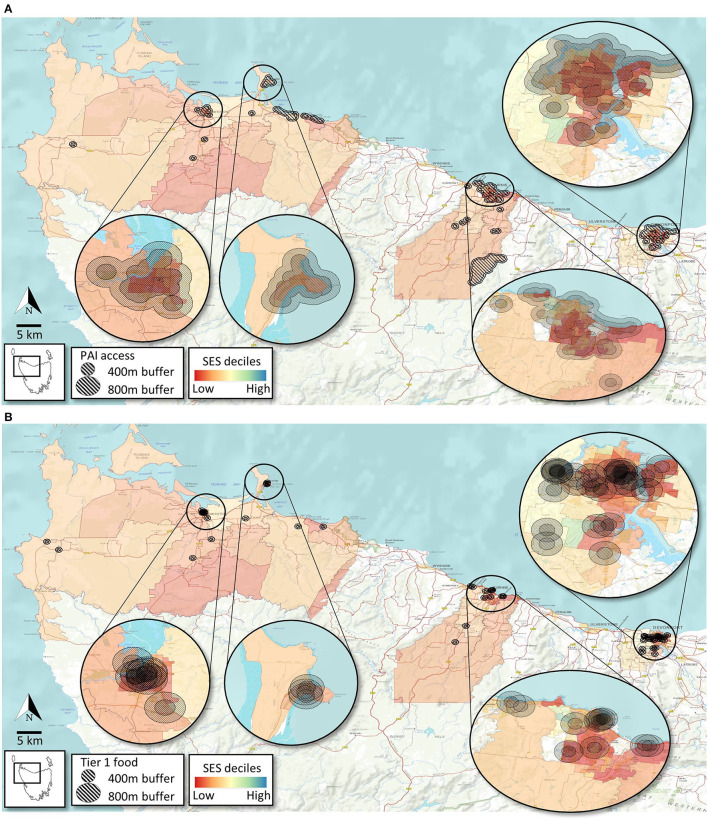
Access to PA infrastructure **(A)** and Tier 1 food outlets **(B)** based on socioeconomic level of suburbs (SA1s) across the study area.

As depicted in Figures 3A,B–in different shades of red and yellow—most of the study area contains suburbs that are socio-economically disadvantaged ( ≤ 6 IRSAD decile). Accessibility to PAI varies markedly amongst different IRSAD areas within and between the sites (Table 4) with pockets of high socio-economic disadvantage (darker shades of red) in all 3 sites having some degree of poor accessibility to PAI (Figure 3A). Overall, when any PAI is considered, only a small proportion (under 30%) of residents in these NW Tasmania sites have poor access (Table 4). However, for a considerable proportion of the population, free-to-access natural amenities/green space and recreational tracks (73 and 57%, respectively) are more than 800 m from their households (Table 4). Some of the suburbs with higher SES (>6 IRSAD percentile) in Burnie and Devonport have exceptionally poor access to natural amenities/green space and recreational tracks (Table 4). The interface between SES and accessibility to “Tier 1 food” also appears to be as varied and complex as PAI, with ~50% (overall) of residents in Burnie, Circular Head and Devonport located >800 m from a healthier “Tier 1” food store. Similar to PAI accessibility, there are pockets of socioeconomically disadvantaged areas in all three study sites that have poor access to Tier 1 food outlets (Figure 3B). Furthermore, ~25 and ~30% of people across the sites have “limited access” (defined as 400–800 m) to PAI and Tier 1 food outlets (Table 5).

**Table 4:** Proportion of the population in each region with poor access to PAI and food outlets based on socioeconomic disadvantage (IRSAD).

	**PAI**				**Recreational tracks**				**Natural amenities and green space**				**Tier 1 Food outlets**			
**IRSAD**	**B**	**CH**	**D**	**SR**	**B**	**CH**	**D**	**SR**	**B**	**CH**	**D**	**SR**	**B**	**CH**	**D**	**SR**
**Dec**														
1	15.34	0.00	11.62	12.42	97.35	9.98	60.60	72.57	12.70	13.40	68.45	42.02	39.24	5.92	13.97	23.88
2	22.53	51.42	1.94	15.91	86.23	81.76	70.70	76.57	19.29	69.00	80.34	62.97	56.11	62.42	32.28	43.63
3	43.90	68.02	6.05	38.82	95.09	73.61	45.20	70.03	46.15	70.41	64.83	61.12	75.37	74.24	70.85	73.38
4	40.12	98.14	54.21	57.48	65.65	99.18	59.54	71.84	43.50	98.38	100.00	71.55	48.97	98.48	43.25	59.00
5	0.00	98.08	62.47	44.19	80.88	99.99	81.13	82.04	0.00	99.28	85.59	58.69	55.49	99.24	92.16	80.70
6	0.00	NA	97.37	47.55	100.00	NA	97.53	98.80	69.55	NA	99.88	84.36	62.39	NA	74.80	68.45
7	0.78	NA	13.30	10.53	100.00	NA	6.13	26.87	47.21	NA	42.61	43.63	95.16	NA	57.40	65.75
8	NA	NA	84.95	84.95	NA	NA	100.00	100.00	NA	NA	100.00	100.00	NA	NA	79.80	79.80
9	5.75	NA	NA	5.75	100.00	NA	NA	100.00	60.22	NA	NA	60.22	38.58	NA	NA	38.58
**Total**	**23.72**	**61.53**	**18.66**	**27.48**	**88.98**	**72.73**	**62.31**	**73.59**	**27.17**	**68.54**	**75.05**	**56.79**	**52.24**	**67.72**	**40.27**	**49.05**

*Poor access is defined as being >800 m from each infrastructure type or food outlet. NA is used when a given region does not have that IRSAD level (1 being the lowest socioeconomic level and 10 is the highest; no SA1s in the study region were in the 10th decile)*.

*Burnie, B; Circular Head, CH; Devonport, D; Study region, SR*.

**Table 5:** Proportion of the population in each region with limited access to PAI and food outlets based on IRSAD.

	**PAI**				**Recreational tracks**				**Natural amenities and green space**				**Tier 1 food outlets**			
**IRSAD**	**B**	**CH**	**D**	**SR**	**B**	**CH**	**D**	**SR**	**B**	**CH**	**D**	**SR**	**B**	**CH**	**D**	**SR**
**Dec**													
1	32.12	3.63	25.44	26.83	2.62	52.71	23.11	16.52	52.45	42.52	21.93	35.80	46.76	57.72	47.37	47.77
2	34.64	8.52	31.74	28.34	8.01	13.59	21.03	16.43	37.79	21.66	11.27	19.78	24.80	24.63	35.65	30.96
3	25.70	17.92	37.60	27.23	4.17	8.93	20.90	11.73	8.86	11.06	19.95	13.54	16.74	15.38	20.24	17.51
4	15.62	1.37	9.14	10.55	17.76	0.67	13.12	12.52	20.16	1.07	0.00	10.27	20.80	1.17	24.83	17.30
5	31.13	1.78	25.25	25.91	15.58	0.01	1.68	6.08	9.26	0.57	8.19	8.13	30.54	0.63	7.79	14.75
6	29.86	NA	2.60	16.54	0.00	NA	1.67	0.82	30.45	NA	0.12	15.64	33.04	NA	17.29	25.35
7	69.72	NA	47.90	52.72	0.00	NA	15.35	11.96	45.89	NA	6.95	15.55	4.84	NA	16.94	14.26
8	NA	NA	15.05	15.05	NA	NA	0.00	0.00	NA	NA	0.00	0.00	NA	NA	14.08	14.08
9	48.63	NA	NA	48.63	0.00	NA	NA	0.00	39.78	NA	NA	39.78	51.59	NA	NA	51.59
**Total**	**29.23**	**10.61**	**27.88**	**25.54**	**6.96**	**13.85**	**18.56**	**13.63**	**33.26**	**15.53**	**14.85**	**21.57**	**31.86**	**20.05**	**32.89**	**30.42**

*Limited access is defined as being between 400 and 800 m from each infrastructure type or food outlet. NA is used when a given region does not have that IRSAD level (1 being the lowest socioeconomic level and 10 is the highest; no SA1s in the study region were in the 10th decile)*.

*Burnie, B; Circular Head, CH; Devonport, D; Study region, SR*.

### Density

**Figure 4:**
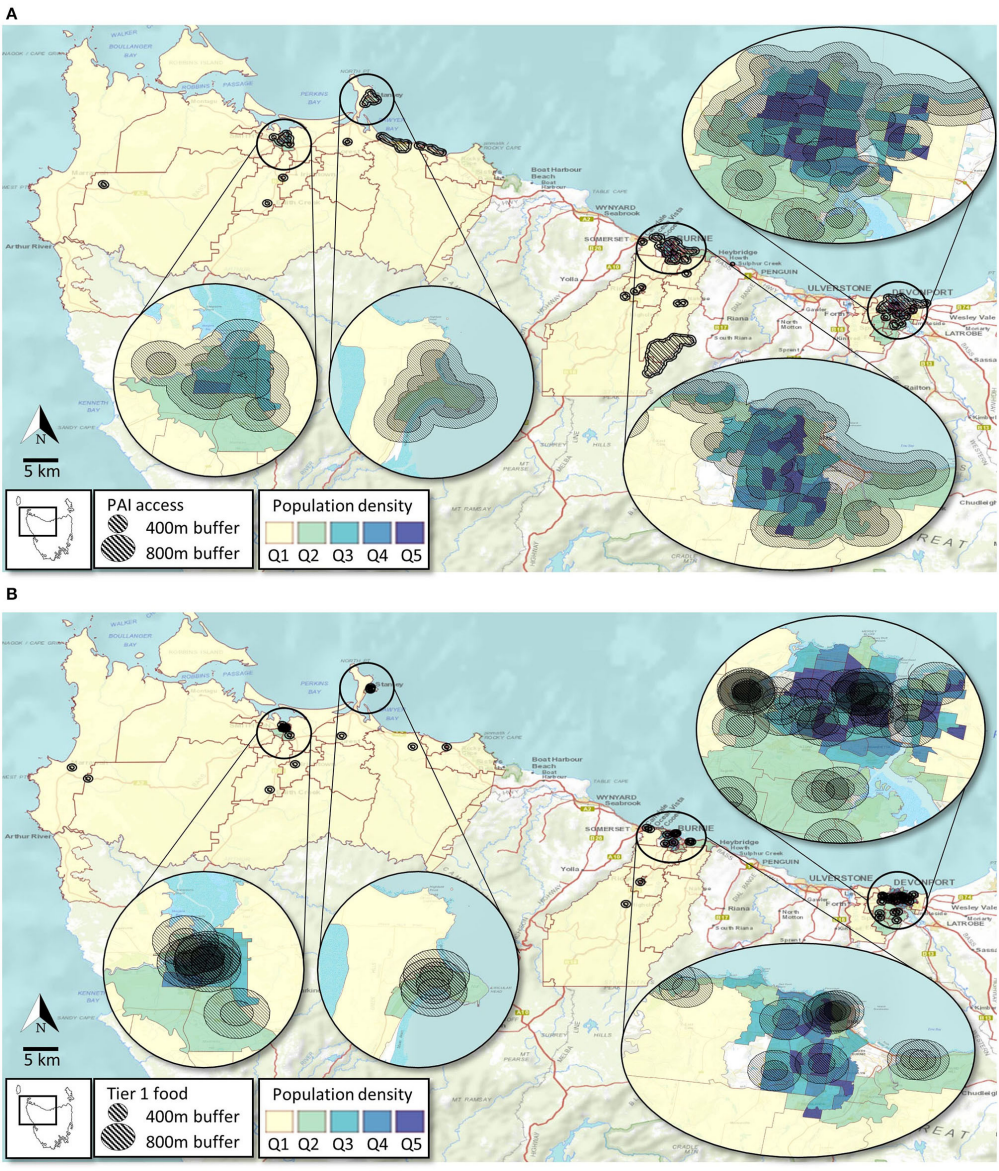
Access to PA infrastructure **(A)** and Tier 1 food outlets **(B)** based on population density of suburbs (technically SA1s) across the study area.

Accessibility of PAI and Tier 1 food outlets by population density is depicted in Figures 4A,B. Areas with the highest population density (i.e., top population density quintiles) have the most access to PAI and Tier 1 food outlets in all three study sites (Table 6 and Figures 4A,B). Nevertheless, accessibility to Tier 1 food outlets appear poor (i.e., >800 m) within some pockets of high population density areas in Burnie, Circular Head, and Devonport.

**Table 6:** Percent of the population with poor access (>800 m) to PAI and healthy food outlets based on population density quintiles across whole study area.

**Population density (people/km^**2**^)**	**PA facilities**	**PA tracks**	**PA reserves**	**Tier 1 food**	**Total area (km^**2**^)**
1–41	97.07	99.13	95.81	98.46	95663.49
41–613	47.52	76.58	65.97	63.37	27481.72
614–1,103	2.00	60.79	40.44	41.47	33156.60
1,104–1,631	9.99	70.28	34.21	38.72	15619.93
>1,631	3.31	68.14	58.65	20.66	7835.02
**Total**	**27.48**	**73.59**	**56.79**	**49.05**	**179756.75**

## Discussion

A considerable body of empirical evidence—with a biassed representation of urban environments—illustrates the associations between lifestyle factors that contribute to obesity (and related chronic diseases), SES, and attributes of the built environment (23–25, 34). Given the pivotal role of “the living environment” on disease incidence and progression, there is a need to better understand the local food and physical activity environment in regional areas where there is often higher obesity-related chronic disease prevalence, as seen in NW Tasmania. Accordingly, this research evaluated access (i.e., coverage, variety, density, and proximity) to physical activity resources and food outlets in relation to SES in three NW Tasmanian communities. Overall, a considerable variety of physical activity resources and food outlets were observed with variability in access across socioeconomic and population density categories.

In the Australian context, poor functionality in available PAI, lack of diverse opportunities to be active, and environmental limitations have been highlighted as pressing concerns leading to sub-optimal levels of physical activity in rural areas (35). Yet the abundance and diversity of PAI found in the study region—including ~25% of them being natural amenities/recreational tracks, and ~20% associated with schools—is intriguing. Perhaps what is lacking are intervention strategies to address barriers to physical activity participation and initiatives that enhance access to extant PAI. Such approaches have been successful in promoting physical activity and reducing sedentary behaviours equitably elsewhere (36). Inherent geographical, economic, and social challenges experienced by rural residents are likely to be significant factors that shape the availability of opportunities to maintain an active lifestyle, compared with those living in urban settings (37–39).

We observed a marked variation in accessibility to PAI amongst different areas of disadvantage within and between the sites with pockets of high socio-economic disadvantage in all three sites having some degree of poor accessibility to facilities. This is in line with previous research, where lack of accessibility/ affordability of PAI and SES are intertwined. More disadvantaged neighbourhoods are disproportionately affected with residents less likely to be able to access or afford recreational facilities and engage in adequate levels of physical activity (14, 15, 40–42). However, in the current study patterns were variable across sites and did not follow a clear SES gradient (Table 4).

Our observations across the study region recorded 47 free-to-access PAI, more than anticipated given the overall SES is lower than the national average (26). Provision of natural amenities/green spaces and recreation tracks (collectively known as public open spaces in some instances), are critical for healthy, sustainable living as has been acknowledged in the United Nations Sustainable Development Goals (43). This is particularly important in the Australian context where housing footprints have increased and space for lawns and gardens have decreased consistently in the last few decades (44, 45). Town planners in Australia usually adhere to “park minimum standards” which are based on population-ratio and/or maximum catchment (distances travelled to gain access) standards (46, 47). However, in all three study sites, the free PAI were more than 800 m (approximately a 10-min walk) from most residences, which may dissuade some residents from frequenting them. Although there is no consensus on the amount of green space required (both in terms of per capita as well as distance), existing literature indicates substantial health benefits (including lower prevalence of obesity) when residences are located within 300–400 metres of green space (48, 49). Our observation that some of the suburbs with higher SES (>6 IRSAD percentile in Burnie and Devonport) have exceptionally poor access to natural amenities/green space and recreational tracks, was unexpected. The spatial distribution of some of the wealthier suburbs (i.e., congregation in peri-urban areas of high real estate value but not necessarily proximate to amenities), may explain this pattern. It is widely acknowledged that availability/ accessibility of these facilities is positively related to recreational activity levels (50, 51), although for effective public health promotion and sustainable reduction of obesity prevalence, a better understanding of preferences and patterns of utilisation of open spaces is required (52).

In addition to providing children with a multitude of physical activity options, the substantial amount of PAI based in schools is a significant value-add to the overall communal PAI availability in the region. Most schools in the study region are centrally located within local neighbourhoods and have a range of gymnasia, playgrounds, sports fields, courts etc. that provide residents with invaluable opportunities (albeit restricted to out of school hours in some instances) to be physically and socially active. Participation in sporting pursuits and related benefits for health and well-being is well-known (53). Organised sport has a prominent place in the Australian identity and is an important element of the social fabric of NW Tasmania (54). As such, the availability of a vast array of sporting facilities (footy ovals, gyms, dance studios etc.) in the wider study area is a significant asset. Even so, based on existing Australian population data—which indicate significantly lower “sport and recreation” participation rates in rural/remote areas compared with inner regional (75.9 vs. 80.8%) and major cities (75.9 vs. 84%)—it is unclear whether availability of facilities translates to physical activity in a significant proportion of the local population (55, 56). Given the availability of substantial sport related infrastructure, there are ample cost-effective and safe opportunities for residents in Burnie, Circular Head, and Devonport to participate in a range of sporting activities in a social setting which could form the basis of health promoting public health initiatives.

In the current study, only a small proportion of the food outlets (~6, 13, and 10% of outlets in Burnie, Circular Head and Devonport, respectively) sold predominantly healthier/perishable foods (i.e., Tier 1 outlets). Similarly, only a small proportion of residents are within a reasonable walking distance (i.e., 5–10 min walk) from them. Food outlets selling predominantly energy-dense, highly processed food (i.e., Tier 2 outlets) were considerably closer to individual dwellings. Accessibility to affordable, fresh, and good-quality food in local neighbourhoods can play an integral role in communal dietary habits (34, 57, 58). Importantly, proximity to perishable food options with high nutrient value (e.g., fresh fruit, vegetables, and lean meat) has been associated with healthier dietary patterns (59). Globally, disadvantaged communities (e.g., low SES or culturally diverse communities) have often been reported to have limited/poor access to these outlets (60–62). As extant evidence indicate, this lack of access (referred to as “food deserts” in some contexts) to healthy food can potentially manifest into obesity related debilitating chronic conditions (63, 64).

The link between low fruit and vegetable availability and increased risk for poor health outcomes is also well-documented (34). Although the precise mechanisms for this relationship are yet to be elucidated, previous reports (including some from Australia) have highlighted elements such as SES-driven purchase habits (e.g., wealthier regions having more purchasing power), demographic factors (e.g., proportion of females) and spatial patterning of food outlets (e.g., wealthier areas having more supermarkets and less fast food outlets) to be important mitigating factors (65–67). Recently, reports have suggested that long-term poverty and welfare dependency are significant contributors to dietary choices (e.g., increased purchase of imported cheap foods with low nutritional value over healthy perishables) of people living in rural Tasmania (68). Given the socio-demographic and geographical layout of NW Tasmania, it is likely that a combination of these factors is at play in different manifestations of the existing food environment.

## Conclusion

Considering the well-established link between poor diet, inadequate physical activity, obesity, and non-communicable diseases in low SES communities, the observed spatial patterns in the current study (i.e., relative ease of access to unhealthier food and moderate access to natural amenities and green space), should be of concern. Overall, although a wide variety of PAI and food options are available to the residents in the study region, a substantial number are beyond an (approximately) 10-min walk from most households, a factor shown to reduce use/visitation rates (69–71). In fact, recent health metrics indicate that lifestyle risk factors such as PA, fruit/vegetable consumption, and sugary beverage consumption have been trending in the wrong direction in the past decade (5). As such, it is possible that the existing layout of food and physical activity environments of Burnie, Circular Head and Devonport are contributing to the obesogenic nature of these communities. Based on our observations, the following actionable strategies are recommended in the NW of Tasmania.

1. Increase public awareness of available PAI—particularly the free-to-access natural amenities and green space.
2. Increase connectivity between PAI resources through provision of better transport options.
3. Nutrition education and increased public awareness of Tier 1 food outlets.

Future public health initiatives geared toward generating environments that are conducive to regular physical activity and accessibility of healthy food will require action at strategic and policy levels involving multiple levels of government; findings from this spatial analysis may add significant value to such initiatives.

## Data Availability Statement

The original contributions presented in the study are included in the article/supplementary material, further inquiries can be directed to the corresponding authors.

## Author Contributions

SJ, EF, DK, RS, and AH: conceptualisation, design of the study, formulating research questions, writing and editing draughts, data collection, and analysis. MK, KP, KA, RH, TH, and NB: conceptualisation, design of the study, review, and editing of the manuscript. All authors contributed to the article and approved the submitted version.

## Funding

This work was funded by a National Health & Medical Research Council (NHMRC) grant as part of the CAPITOL Project. The study funder had no role in study design, collection, analysis or interpretation of the data, in writing the report, or in the decision to submit the article for publication. The contents of this article are the responsibility of the authors and do not reflect the views of the NHMRC.

## Conflict of Interest

The authors declare that the research was conducted in the absence of any commercial or financial relationships that could be construed as a potential conflict of interest.

## Publisher's Note

All claims expressed in this article are solely those of the authors and do not necessarily represent those of their affiliated organizations, or those of the publisher, the editors and the reviewers. Any product that may be evaluated in this article, or claim that may be made by its manufacturer, is not guaranteed or endorsed by the publisher.
